# Supplementary material for: Increased Asian aerosols drive a slowdown of Atlantic Meridional Overturning Circulation
Source: Nat Commun. 2024 Jan 2;15:18. doi: 10.1038/s41467-023-44597-x (PMC10762259; doi:10.1038/s41467-023-44597-x)
Supplement: Supplementary file 1 — Supplementary Information [file 41467_2023_44597_MOESM1_ESM.pdf]

Supplementary Information

Increased Asian aerosols drive a slowdown of Atlantic Meridional Overturning Circulation

Fukai Liu[1*†], Xun Li[1†], Yiyong Luo[1*], Wenju Cai[1,2,3,4], Jian Lu[5], Xiao-Tong Zheng[1], Sarah M. Kang[6], Hai Wang[1], Lei Zhou[7,8]

1. Frontiers Science Center for Deep Ocean Multispheres and Earth System, Physical Oceanography Laboratory, and Sanya Oceanographic Institution, Ocean University of China, China
2. Laoshan Laboratory, Qingdao, China
3. State Key Laboratory of Marine Environmental Science & College of Ocean and Earth Sciences, Xiamen University, Xiamen, China
4. State Key Laboratory of Loess and Quaternary Geology, Institute of Earth Environment, Chinese Academy of Sciences, Xi'an, China
5. Atmosphere, Climate, and Earth Sciences Division, Pacific Northwest National Laboratory, Richland, USA
6. Max Planck Institute for Meteorology, Hamburg, Germany
7. School of Oceanography, Shanghai Jiao Tong University, Shanghai, China
8. Southern Marine Science and Engineering Guangdong Laboratory (Zhuhai), Zhuhai, China

[†] These authors contributed equally to this work

Correspondence to: Fukai Liu and Yiyong Luo

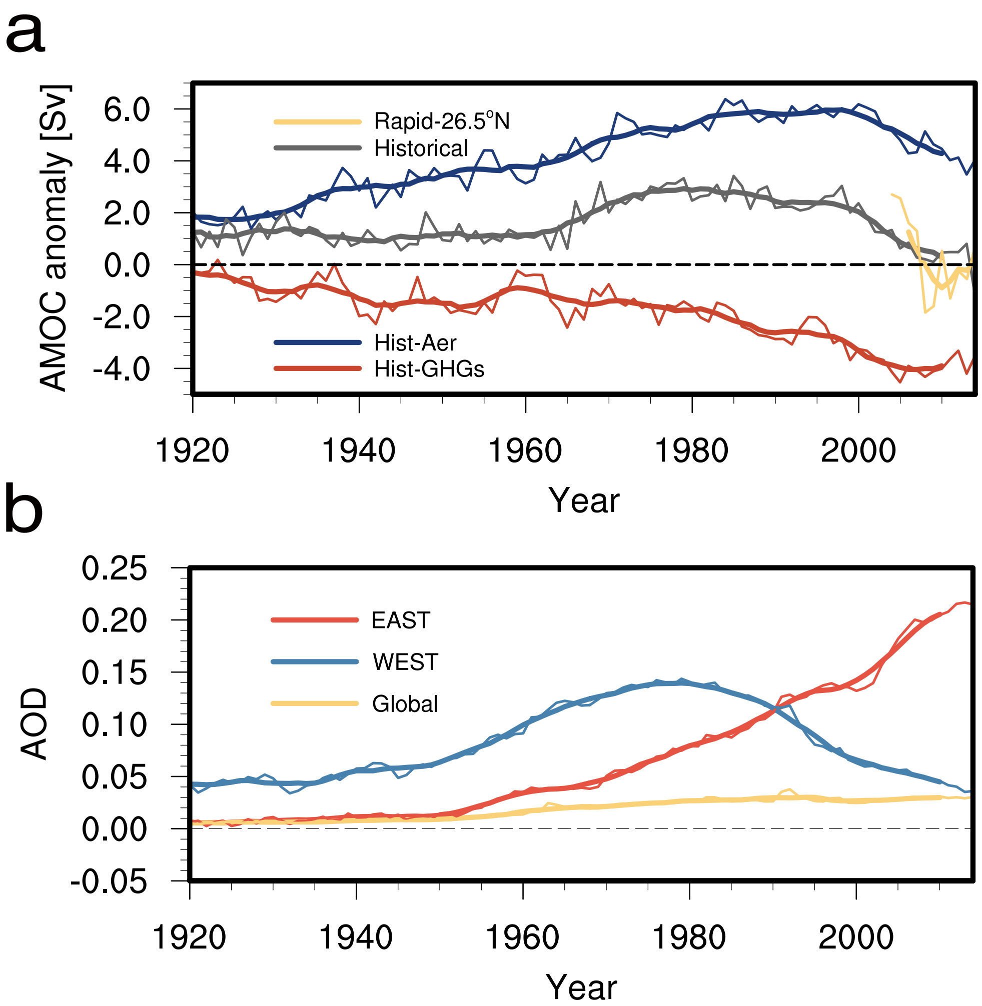

Supplementary Fig. 1 | Changes in AMOC strength and AAs emissions in historical simulations. a, Annual-mean AMOC time series (unit: Sv) in CMIP6 historical experiment (gray lines), DAMIP GHG-alone (red lines) and aerosol-alone experiments (blue lines), and the RAPID observation at 26° N (yellow lines). **b**, The temporal evolution of annual-mean aerosol optical depth (AOD) at 550nm (unit: 1) over the eastern hemisphere (averaged over the red boxed region in Fig. 1a; red lines), the western hemisphere (averaged over the blue boxed region in Fig. 1a; blue lines), and the global mean (yellow lines). In **a** and **b**, the thin lines are the annual means, and the thick lines are the 9-year running means. The model simulated AMOC index in **a** is obtained as the maximum of streamfunction between latitudes 25° N–75° N and between depths of 500 and 2000 m. Since the 1980s, reduced AAs in North America and Europe are believed to contribute to the AMOC slowdown, but it is unclear how increased AAs in Asia affect the AMOC.

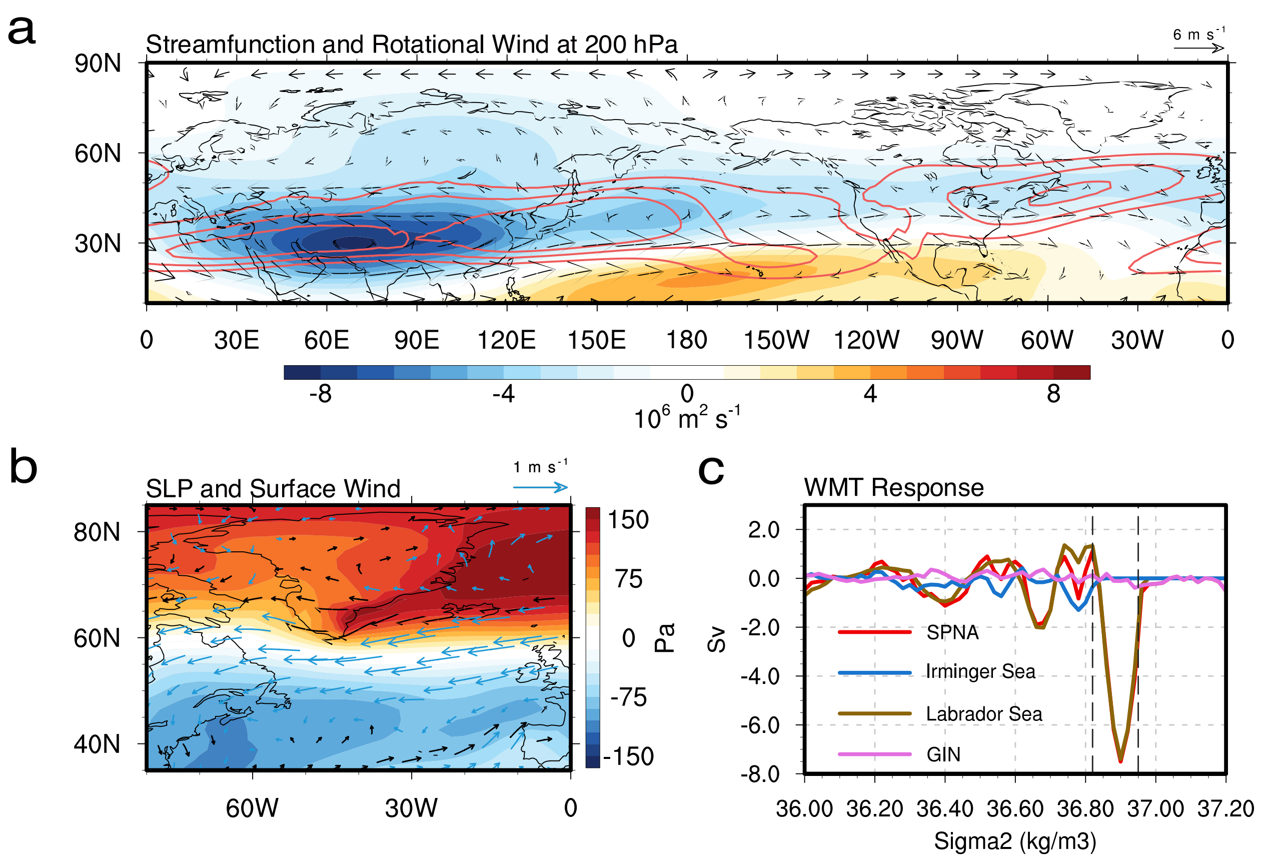

Supplementary Fig. 2 | Simulated long-term responses to AAs changes. a-c, Same as Fig. 2b, 2c and 3b, respectively, but averaged over model years 51-100. The long-term responses of atmospheric circulation and water mass transformation show consistent patterns compared to the transient responses.

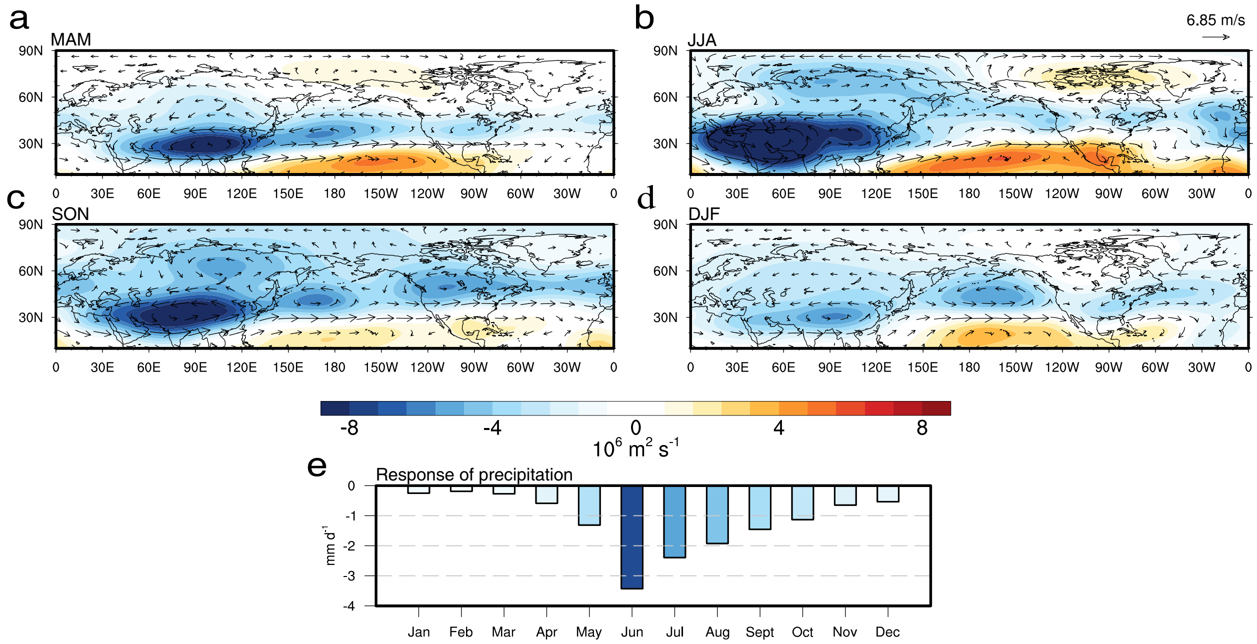

Supplementary Fig. 3| Seasonality of the teleconnection pattern to increased Asian AAs. a-d, Responses of seasonal-mean 200-hPa streamfunction (colors; unit: $10^6\ m^2\ s^{-1}$) and rotaintional wind (vectors; unit: $m\ s^{-1}$) in EAST. **e,** Responses of monthly-mean precipitation (colors; unit: $mm\ day^{-1}$) averaged over the South and East Asia (red boxed region) in Fig. 1a. The circumglobal wave pattern triggered by increased Asian AAs persists throughout the year and peaks in summer, likely due to the stronger convective feedback to external radiative forcing during the active Asian Monsoon season.

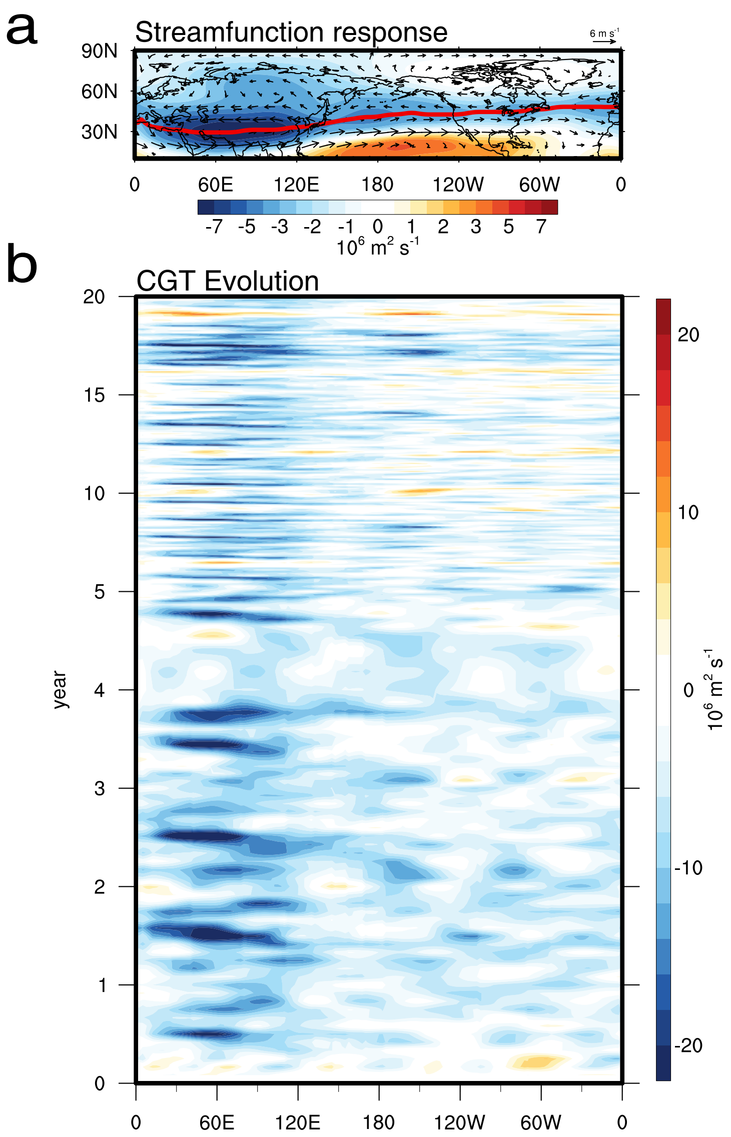

Supplementary Fig. 4 | Temporal evolution of the teleconnection pattern excited by increased Asian AAs. a, Response of annual-mean 200-hPa streamfunction (colors; unit: $10^6\ m^2\ s^{-1}$), the red line denotes the mean path of the quasi-stationary wave train, which is defined as the latitudinal position of the maximum anomalies. **b**, Temporal evolution of monthly-mean 200-hPa streamfunction along the mean path of the quasi-stationary wave (red line in **a**). The circumglobal response emerges within the first few months, suggesting a rapid influence of Asian AAs on the atmospheric teleconnection pattern.

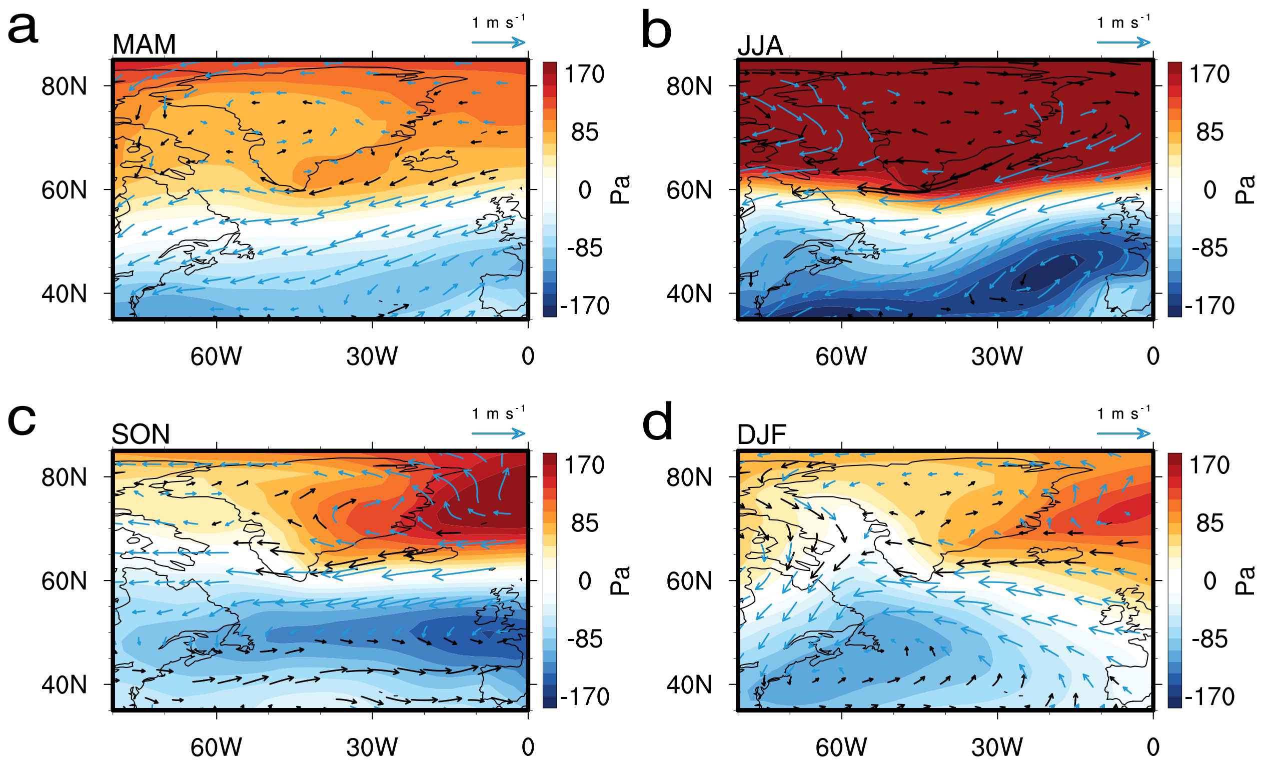

Supplementary Fig. 5| Seasonality of the negative-NAO like response to increased Asian AAs. a-d, Responses of seasonal mean sea level pressure (colors; unit: Pa) and surface wind (vector; unit: $m\ s^{-1}$) over the North Atlantic. Blue and black vectors denote anomalous winds that weaken and intensify the climatological winds, respectively. The negative NAO-like response triggered by increased Asian AAs persists throughout the year, leading to suppressed surface winds over the Labrador Sea.

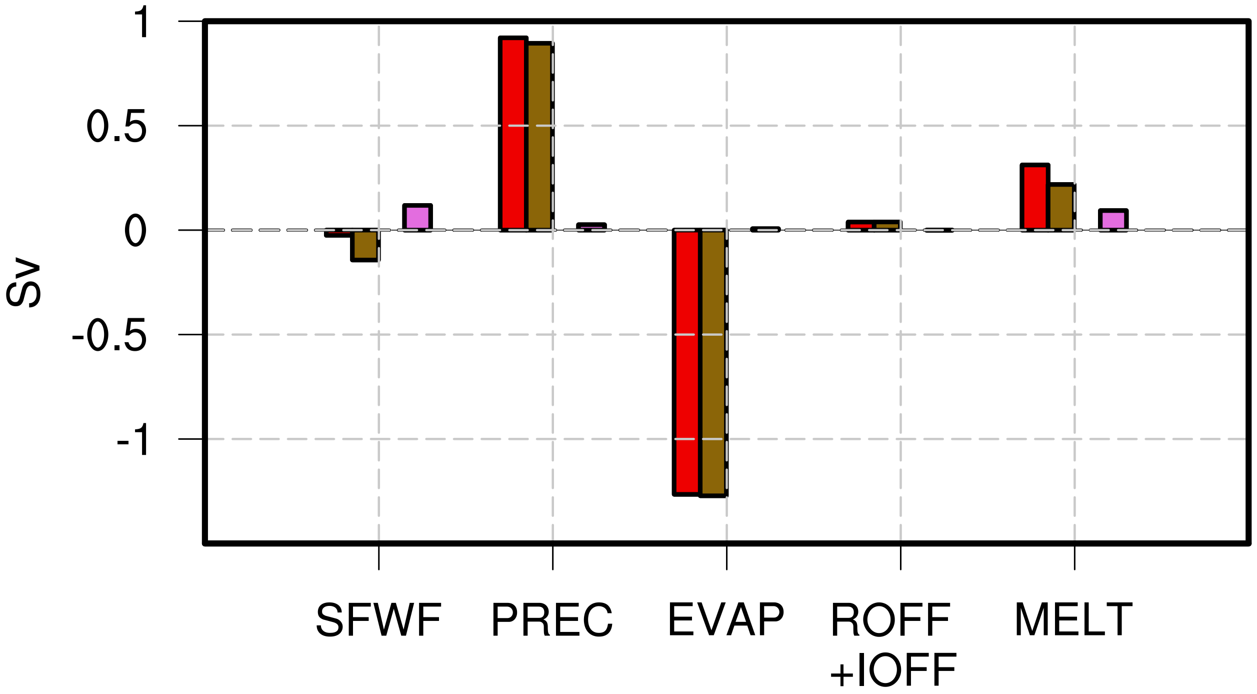

Supplementary Fig. 6 | Haline contribution to the WMT reduction in the subpolar North Atlantic. The same as Fig. 3c, but for the saline component (SFWF), and its decomposition into precipitation (PREC), evaporation (EVAP), liquid and solid runoff (ROFF+IOFF), and melting flux (MELT). The haline contribution to the WMT reduction is negligible compared to the thermal contribution.

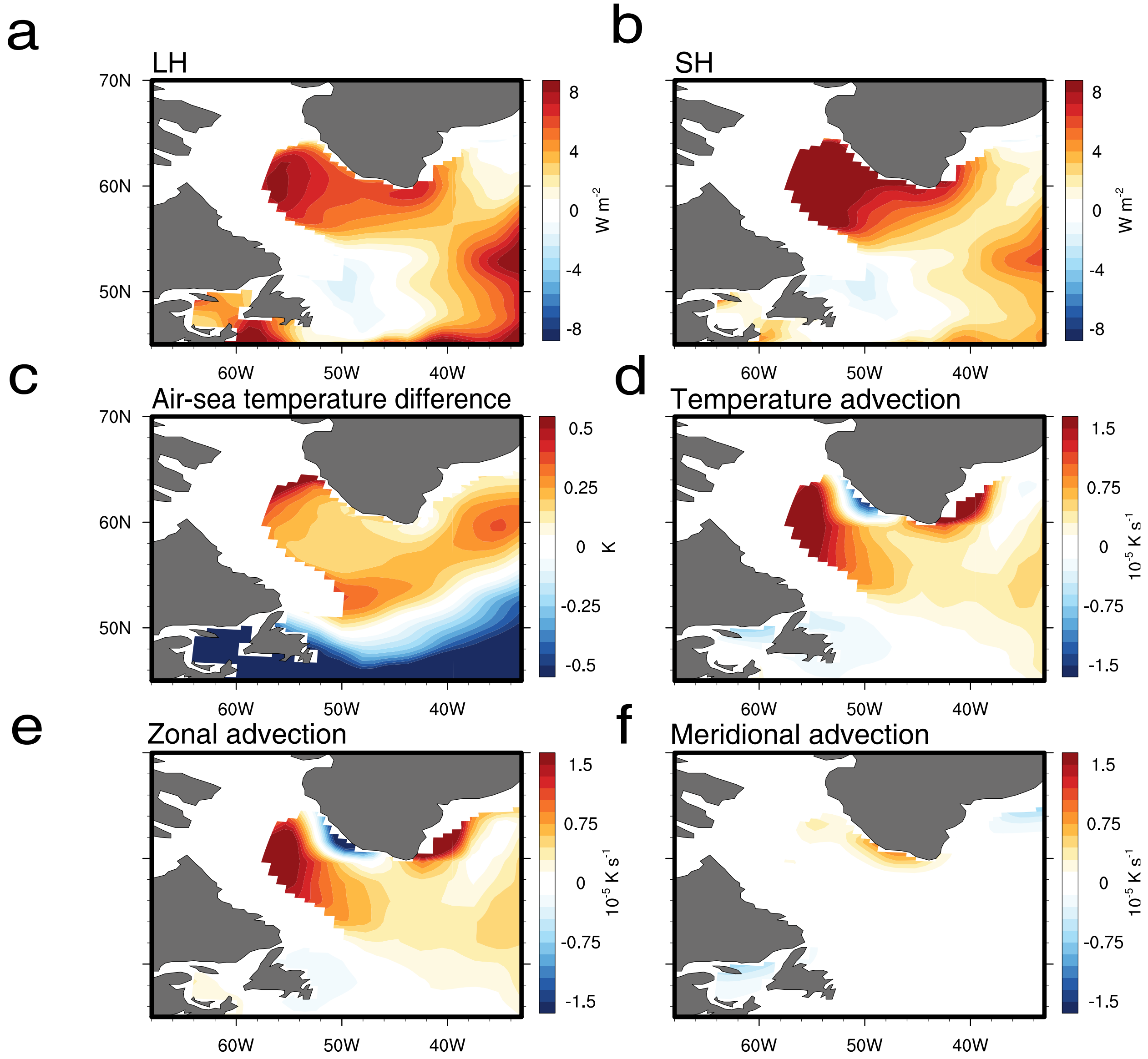

Supplementary Fig. 7 | Responses of air-sea interface variables over the subpolar North Atlantic to increased Asian AAs. a, Latent heat flux (unit: $W\ m^{-2}$; positive downward). **b,** Sensible heat flux (unit: $W\ m^{-2}$; positive downward). **c**, Air-sea temperature difference (unit: K). **d-f**, Atmospheric temperature advection (unit: K m/s) and its decomposition into zonal and meridional components. Positive values in **c** indicate a decreased air-sea temperature difference. Regions where sea ice concentrations are greater than 5% are masked out. Suppressed westerlies reduce cold air transport from North America, reducing air-sea temperature difference and hindering turbulent heat loss in the Labrador Sea.

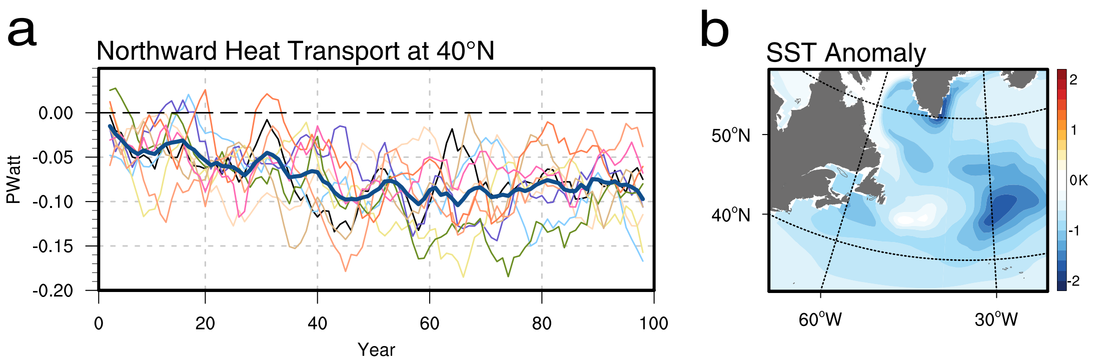

Supplementary Fig. 8 | The decreased northward heat transport mitigating the rate of AMOC weakening. a. Ensemble-mean evolution of oceanic northward heat transport at 40ºN (unit: PW) in EAST smoothed with a 5-yr running-mean filter, with the thin curves denoting each member's anomalies. **b**, SST response (unit: °C) in EAST averaged over year 51-100. The weakened AMOC reduces the northward oceanic heat transport and advects anomalously cold water into the deep convection site, balancing the surface heat gain there.

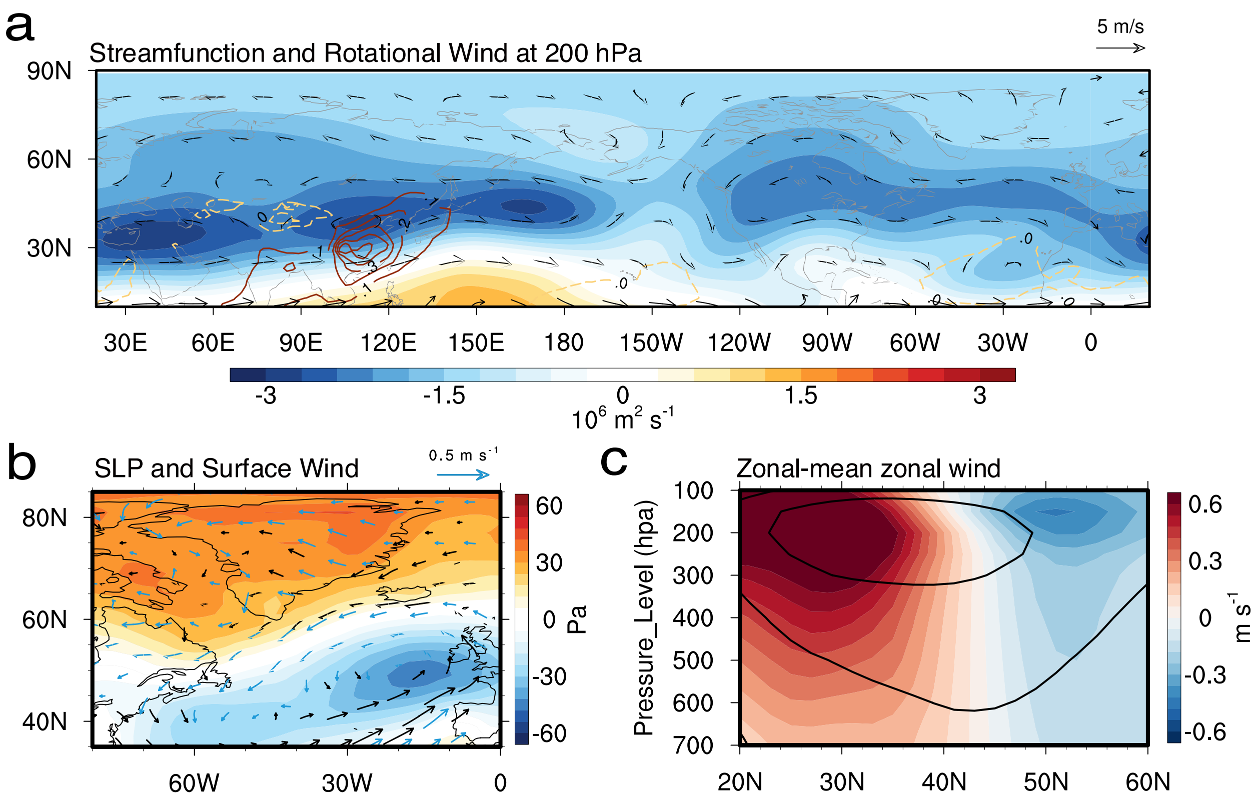

Supplementary Fig. 9 | Responses in atmospheric circulation to 2014 sulfate emission forcing in AerChemMIP simulations. a, Responses of aerosol optical depth (contours; unit: 1), 200-hPa streamfunction (colors; unit: $10^6\ m^2\ s^{-1}$) and rotational wind (vectors; unit: $m\ s^{-1}$) calculated as the difference between the piClim-SO2 ensemble and the piClim-control ensemble for years 1-30. **b**, Responses of sea level pressure (colors; unit: Pa) and surface wind (vector; unit: $m\ s^{-1}$) over the North Atlantic from the multi-model mean. Blue and black vectors denote anomalous winds that weaken and intensify the climatological winds, respectively. **c**, Response of the zonal-mean zonal winds (colors; unit: $m\ s^{-1}$) from the multi-model mean, and superimposed is the corresponding climatology in piClim-control. The Asian AAs-induced atmospheric responses are validated through the AerChemMIP simulations.

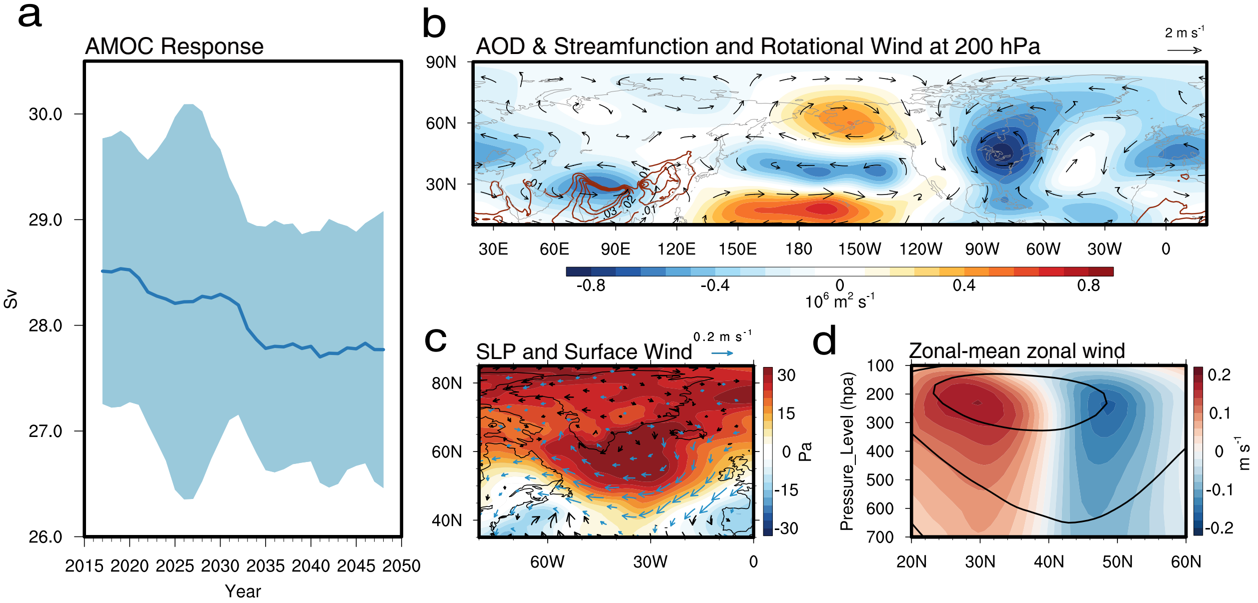

Supplementary Fig. 10 | Responses in atmospheric circulation and AMOC to increasing Asian AAs in CESM2-SF-LE simulations. a, AMOC time series (unit: Sv). The AMOC index is obtained as the maximum of streamfunction between latitudes 25° N–75° N and between depths of 500 and 2000 m. **b-d**, Same as Supplementary Fig. 9a-c, but for the difference between years 2040-2050 and years 2015-2025 of the ensemble mean from SSP370-AAs simulations. The Asian AAs-AMOC link is validated through the CESM2-SF-LE simulations.

Supplementary Table. 1 | The CMIP6 model variables used in this study.

CMIP6 Model	Historical	Hist-GHG<br>Hist-AAs	piClim-control<br>piClim-SO2
ACCESS-CM2	msftmz, od550aer, ua, va, slp	msftmz	
ACCESS-ESM1-5	msftmz, od550aer	msftmz	
BCC-ESM1			od550aer, ua, va, slp
CanESM5	msftmz, od550aer	msftmz	
CESM2	msftmz, od550aer	msftmz	
CESM2-FV2	od550aer		
CMCC-ESM2	od550aer		
CNRM-ESM2-1			od550aer, ua, va, slp
E3SM-1-0	od550aer		
E3SM-1-1-ECA	od550aer		
E3SM-2-0	od550aer		
EC-Earth3-AerChem			od550aer, ua, va, slp
EC-Earth3-Veg	od550aer		
GFDL-ESM4	od550aer		od550aer, ua, va, slp
GISS-E2-1-G			od550aer, ua, va, slp
INM-CM4-8	od550aer		
INM-CM5-0	od550aer		
IPSL-CM6A-LR	od550aer		
IPSL-CM6A-LR-INCA			od550aer, ua, va, slp
MIROC6	od550aer		od550aer, ua, va, slp
MPI-ESM-1-2-HAM	od550aer		od550aer, ua, va, slp
MPI-ESM1-2-HR	od550aer		
MRI-ESM2-0	msftmz, od550aer	msftmz	od550aer, ua, va, slp
NorESM2-LM	msftmz, od550aer	msftmz	od550aer, ua, va, slp
NorESM2-MM	od550aer		
UKESM1-0-LL			od550aer, ua, va, slp
FGOALS-g3	msftmz, od550aer	msftmz	

"msftmz" denotes the ocean overturning mass streamfunction; "od550aer" denotes the aerosol optical depth at 550nm, "ua" and "va" denote the zonal and meridional wind speed, and "psl" denotes the sea level pressure, respectively.
